# Professional quality of life and job satisfaction among nurses working at tertiary hospitals in central Ethiopia

**DOI:** 10.1186/s12912-024-02101-w

**Published:** 2024-06-21

**Authors:** Sentayehu Admasu Saliya, Taye Mezgebu Ashine, Asnakech Zekiwos Heliso, Getachew Ossabo Babore, Bethelhem Birhanu, Awoke Girma Hailu

**Affiliations:** College of Medicine and Health Science, School of Nursing, Wachemo University, Hosanna, Ethiopia

**Keywords:** Professional quality of life, Compassion fatigue, Compassion satisfaction, Job satisfaction

## Abstract

**Background:**

Professional quality of life is a crucial aspect of healthcare professionals’ well-being and job satisfaction. Job satisfaction, on the other hand, encompasses fulfillment of desired needs within the work environment, happiness or gratifying emotional response towards working conditions, and job values or equity. Existing literature tends to address job satisfaction and professional quality of life separately, overlooking their interconnectedness, especially within the unique context of Ethiopia. This study aimed to assess nurses’ professional quality of life and job satisfaction.

**Methods:**

A descriptive cross-sectional study was conducted from September 1–30 2023 among 420 nurses using a structured questionnaire. The study participants were recruited by simple random sampling. Multiple linear regressions were used to identify factors associated with outcome variables.

**Results:**

The study involved 420 nurses, with 407 completing the questionnaire, yielding a 96.68% response rate. The findings revealed varying levels of professional quality of life. Specifically, 258 participants (63.4%) exhibited low compassion satisfaction, while 271 (66.6%) and 266 (65.4%) experienced average levels of burnout and secondary traumatic stress, respectively. Job satisfaction was moderate to high for 55% of the participants. As the finding of this study indicates, there is a positive correlation between compassion satisfaction and job satisfaction in nursing. The study also identified predictors for job satisfaction, compassion satisfaction, and compassion fatigue, such as marital status, education, and experience.

**Conclusion:**

The majority of participants reported a medium level of compassion satisfaction, with a significant proportion experiencing moderate to high levels of compassion fatigue. Although more than half of the participants had moderate to high job satisfaction, there were still low levels of satisfaction. The study recommends developing targeted training programs, implementing workplace policies, and designing initiatives to enhance education, experience, and compassion satisfaction.

## Introduction

The standard of patient care and the well-being of healthcare professionals are significantly impacted by professional quality of life and job satisfaction in the nursing profession [1].

A nurse’s job-related satisfaction is impacted by both their favorable and detrimental daily work experiences [2]. Compassion Satisfaction is a positive aspect of professional quality of life, whereas Compassion Fatigue is a negative aspect. Two factors contribute to compassion fatigue. Burnout, which includes tiredness, frustration, rage, and despair that are usually connected to one’s job, is the first element. The second element is secondary trauma stress, which is an uncomfortable feeling brought on by fear and trauma at work [3].

In the nursing field, job satisfaction was characterized by three aspects: the ability to meet needs in the workplace, happiness or a positive emotional reaction to working conditions, and employment equity or values [4].

In the nursing context, these two elements are intricately interrelated, as the emotional demands and stressors inherent in the profession directly impact the overall job satisfaction of nurses [5]. The emotional toll of empathetic caregiving can have a significant impact on a nurse’s general well-being. Excessive compassion fatigue can have negative effects on one’s physical and mental health, while high levels of compassion satisfaction are linked to job satisfaction and general well-being [6].

Burnout, compassion fatigue, and workload pressures are among the key contributors to diminished professional quality of life and job satisfaction in nursing [7]. The study from the United States indicates compassion satisfaction is positively correlated with job satisfaction [8]. Studies have indicated that individuals who provide support to others who have had traumatic stressors may be more susceptible to negative symptoms of depression, burnout, and post-traumatic stress disorder [9–12]. On the other hand high level of job satisfaction has a positive effect on a high level of professional quality [13]. Job satisfaction is crucial to prevent burnout. It is defined as a “syndrome resulting from chronic workplace stress that was not properly addressed” [14].

In Ethiopia, the national pooled prevalence of job satisfaction among health professionals is 46.17% [15], However, there is a significant variation in job satisfaction levels across different regions of the country. For instance, the study conducted in the Amhara region reported a high level of job satisfaction among health professionals, with a mean score of 57.5 [16] On the other hand, a cross-sectional study in the Oromia region found that only 16.5% of health professionals were satisfied with their jobs.

In contrast, studies have reported moderate levels of compassion fatigue among health professionals in Ethiopia [17]. Similarly, a study conducted in South Africa, Turkey Saudi Arabia, and the United States reported moderate levels of compassion fatigue among healthcare providers [10, 11, 18, 19]. Studies from Latvia indicate there was a positive correlation between compassion fatigue and secondary traumatic stress [20].

Achieving the best possible patient outcomes depends on nursing practitioners who feel satisfied with their work [1]. A significant positive relationship was observed between compassion satisfaction and clinical competence [21]. Nurses with higher levels of compassion satisfaction and lower levels of compassion fatigue and secondary traumatic stress tend to have higher levels of clinical competencies [22].

Altruism and the satisfaction that comes from being able to help others might be seen as the positive sides of helping. The degree of the painful content that the caregiver is exposed to such as direct contact with victims exacerbates the negative impacts of caregiving, especially when the exposure is ugly and explicit. Burnout, depression, increased substance use, and symptoms of posttraumatic stress disorder are possible effects [9].

Despite the abundance of individual studies on job satisfaction [15, 16, 23, 24] and professional quality of life [17, 25] in various occupational sectors in Ethiopia, there is a lack of comprehensive exploration that consolidates these two critical dimensions into a single study.

The existing body of literature tends to address job satisfaction and professional quality of life separately, overlooking the interconnectedness between these facets, especially within the unique context of Ethiopia, especially in nursing. This gap highlights the need for a comprehensive investigation that not only identifies the distinct contributors to job satisfaction and professional quality of life but also explores their synergies and interdependencies within the Ethiopian work environment. A study was conducted to assess professional quality of life and job satisfaction among nurses working in tertiary hospitals in central Ethiopia.

Objective To assess nurses’ professional quality of life and level of job satisfaction among nurses working at tertiary hospitals in central Ethiopia in 2023.

## Methods

### Study design and period

The institution-based cross-sectional was conducted from September 1–30, 2023.

### Study area

The study was carried out in tertiary-level hospitals in central, Ethiopia. The region is organized into 6 zones and three special woredas on an administrative level. In the region, there are 3 tertiary-level hospitals; Wolikite University Hospital, Wachemo University Hospital, and Worabe Hospital. About 539 nurses were working in those hospitals. Worabe Hospital is located 172 km south of Ethiopia’s capital city, Addis Ababa, in Worabe, a town. There are 112 nurses in the hospital at Worabe Hospital. Wachemo University Hospital Referral is in Hossaena town, 232 km south of Addis Ababa, Ethiopia’s capital city. There are 287 nurses in total at the Wachemo University Hospital. Wolikite University Hospital is located 158 km from Addis Ababa, Ethiopia’s capital. There are 140 nurses in total at the Wolikite University Hospital.

### Population

The source population was all nurses working at tertiary level hospitals of central Ethiopia and the study population was all sampled nurses who were working at tertiary level hospitals in central Ethiopia, 2023.

#### Eligibility criteria

All nurses who were working at tertiary-level hospitals in central Ethiopia, in 2023 were included in the study and nurses who were sick, unable to respond, and on annual leave were excluded from the study.

### Sample size determination

The sample size was calculated using a single population proportion calculation that takes into account the following factors: 95% confidence interval, 46.68% proportion [26], and a margin of error of 5%.

$$N = \frac{{\left( {z\frac{\alpha }{2}} \right)2 \times P\left( {1 - P} \right)}}{{d2}}$$

$N = (1.96)2 * \frac{{0.4801(1 - .0.4801)}}{{0.05 * 0.05}}$ = 382 a total of 420 is obtained by adding a 10% non-response rate.

### Sampling technique

**Fig. 1:**
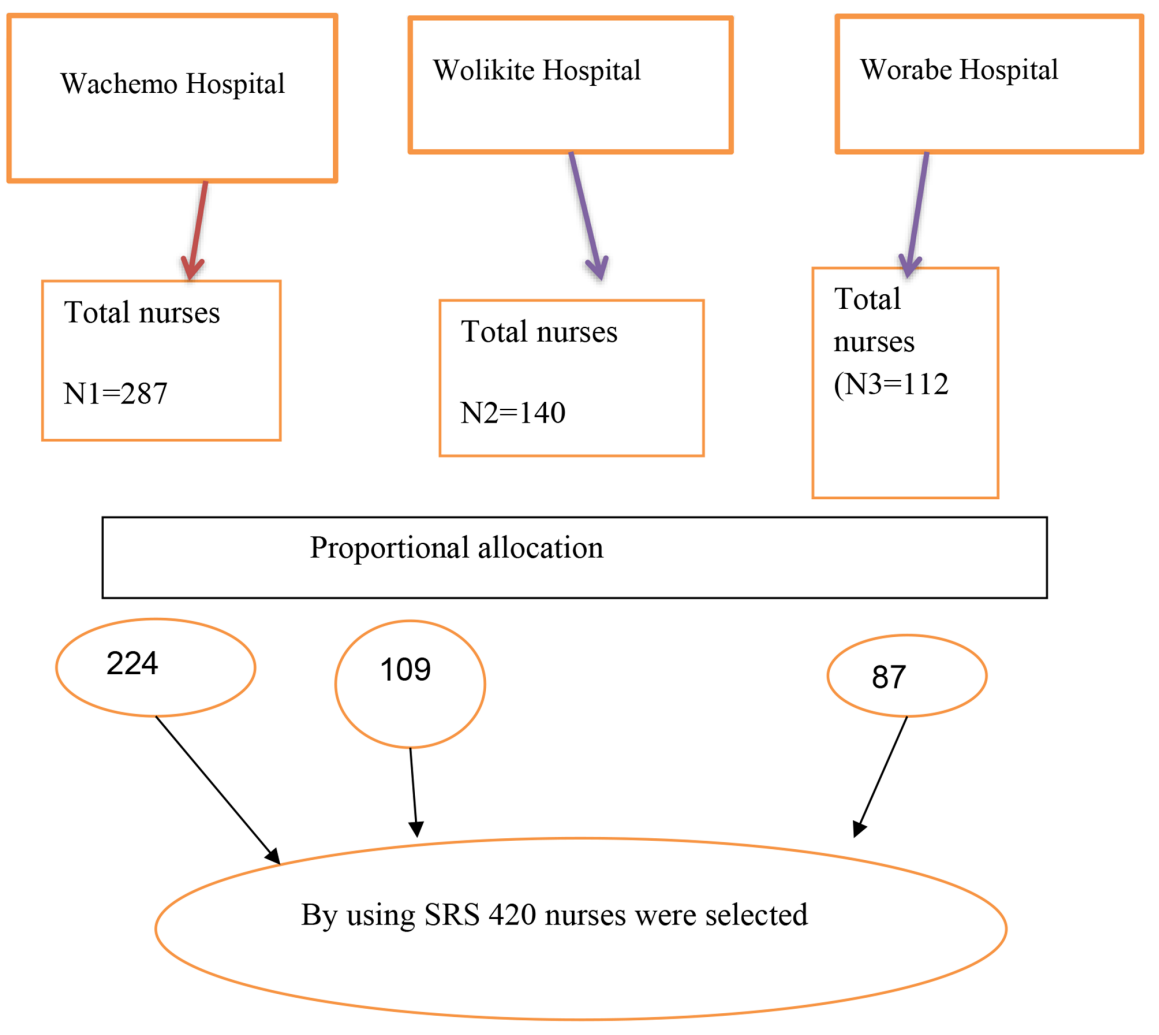
Sampling procedure to select study participants from tertiary level Hospitals in central, Ethiopia 2023

The total sample size was proportionally distributed based on the number of nurses working in each academic tertiary hospital as shown in the figure below (Fig. 1).

$$Ni=\frac{Nh}{Nt}*N$$

Where.

Ni = proportional sample size for each hospital.

Nh = total number of nurses in each hospital.

Nt = overall number of nurses in the hospital.

N = estimated sample size.

Then, the sampling frame was prepared for each hospital by having lists of nurses from the hospital’s human resource management. Finally, nurses of each hospital were selected by a simple random sampling technique using a computer-generated random number from the sampling frame.

### Data collection tool and procedure

Data were collected using pre-tested, structured, and self-administered questionnaires which were adopted from previous Ethiopian studies [24, 27, 28]. The questionnaire was written in English and then translated into Amharic, with questions retranslated back into English for consistency. The questionnaire was divided into socio-demographic, Minnesota Satisfaction scale, and professional quality of life scale.

A pre-test was conducted in Hawassa University’s Comprehensive Specialized Hospital two weeks before the actual data collection time on 10%(42 nurses) of the sample size.

The Minnesota Satisfaction Questionnaire (MSQ) short form was used to assess job satisfaction [29–31]. Twenty questions were rated on a 5-point Likert scale, with 1 signifying severely dissatisfied and 5 denoting very satisfied. Negative items were reversed to positive before summing. The measuring items on nurses’ job satisfaction were achievement, advancement, work itself, recognition, growth at work, organization policy, relationship with colleagues and supervisor, payment, and working conditions. The overall score of the questionnaire ranges from 20 to 100 so the score ranges of 20–47, 48–76, and 77–100 indicate a low, moderate, and high level of job satisfaction, respectively [32].

The reliability test in the previous study revealed that the tool for the subscale was reliable, with a Cronbach’s alpha score of 0.83 [14], in this study reliability test value was acceptable with Cronbach’s alpha of 0.891. The mean score was calculated after checking the normality of the distribution.

The professional quality of life scale contains 30 items and the scale has 3 sub-scales [33]: Compassion satisfaction, secondary traumatic stress, and burnout scale. The secondary traumatic stress subscale and burnout subscale measure compassion fatigue. The scale was measured using a point Likert scale (5 = very often to 1 = never) [34]. The higher the score of compassion satisfaction, the higher the compassion satisfaction, the higher the score of compassion fatigue, and the higher the risk of compassion fatigue.

### Dependent and independent variables of the study

#### Dependent variable

- Job Satisfaction.
- Professional quality of life.

### Independent variables

- Socio-demographic factors.
- Age
- Sex
- Educational level
- marital status
- work experience
- income

### Operational definition

#### Job satisfaction

The questionnaire’s total score ranges from 20 to 100, with scores in the three categories of 20–47, 48–76, and 77–100, representing low, moderate, and high levels of job satisfaction, respectively [32].

#### Burnout

A score of 22 or less indicated a low degree of burnout, a score between 23 and 41 indicated an intermediate level of burnout, and a score of 42 or above indicated a severe level of burnout [35].

#### Secondary traumatic stress

A score of 22 or a low level; a score between 23 and 41 an average level, and a score of 42 or more indicated a high level of Secondary traumatic stress [35].

#### Compassion satisfaction

A score of 22 or less low level, a between 22 and 41 is an average level, and a score of 42 or more indicates a high level of Compassion satisfaction [35].

### Data management and analysis

After the data were checked for consistency and completeness, data were entered into EpiData version 4.6 and exported to SPSS (Statistical Package for Social Sciences) version 26 for analysis. Tables, graphs, and charts were used to interpret and show the results. Those that were associated with outcome variables in bivariate analysis at *P* values of 0.25 or lower were included in the multivariable linear regression model. We then performed tests for multicollinearity, including variance inflation factor reports and condition number tests because of the potentially high overlap between the explanatory variables; for example, age and work experience, and gender and profession. Age and years of work experience variables were highly collinear and age was removed from explanatory variables. We report regression coefficients for these models. Variables with estimated coefficients that had *P* values ≤ 0.05 were considered statistically significant associated factors in this study.

### Ethical consideration

Ethical clearance was obtained from the Wachemo University College of Medicine and Health Science, with reference number IRB/172/16. Written informed consent was approved by the Wachemo University College of Medicine and Health Science Institutional Review Board. Written informed consent was obtained from all study participants. To protect participants from risks, the study did not record participants’ names, identification numbers, and names of health facilities where he or she worked.

## Result

### Socio-demographic data

The questionnaires were distributed to the 420 sampled nurses working in tertiary-level hospitals. Four hundred-seven (407) nurses returned the questionnaires, indicating a 96.9% response rate. The participants were between 20 and 41 years old, with a mean age of 30.85 and an SD of 6.39. The respondents predominantly were between the ages of 25 to 29 years. The majority of 220 (54.1%) respondents were female. In terms of marital status, 204 (50.1%) study participants were single. Most respondents, 298 (73.2%), had a BSc degree in nursing. The majority of study participants 248 (60.9%) had less than five years of work experience. The average monthly income of respondents was 888.43, with a minimum of 5000 and a maximum income of 13,000 Ethiopian Birr (Table 1).

**Table 1:** Socio-demographic characteristics of nurses working at tertiary level hospitals in central Ethiopia, 2023

Variables		Frequency	*N* %
Age	20–24	59	14.5%
	25–29	156	38.3%
	30–34	86	21.1%
	≥ 35	106	26.0%
Sex	Male	187	45.9%
	Female	220	54.1%
Marital status	Single	204	50.1%
	Ever married	203	49.9%
Educational level:	Diploma	74	17.4%
	BSc	298	73.2%
	MSc	38	9.3%
Experience in a year	< 5	248	60.9%
	5–10	78	19.2%
	> 10	81	19.9%
Income	<=6200	26	6.4%
	6201–8017	197	48.4%
	>=8018	184	45.2%

### Professional quality of life

The mean compassion satisfaction score among study participants was found to be 26.34 ± 9.38, with a minimum score of 10.00 and a maximum score of 47.00 [1]. On the other hand, the mean compassion fatigue score was significantly higher at 55.26 ± 18.80, with a minimum score of 20.00 and a maximum score of 93.00.

The results suggest that the average mean score of compassion satisfaction was lower than the mean score of compassion fatigue, indicating that most of the study participants experienced lower compassion satisfaction. This finding is concerning, as compassion satisfaction is an essential factor in maintaining the well-being and mental health of healthcare providers see (Table 2).

**Table 2:** Professional quality of life characteristics of nurses working at tertiary level hospitals in central Ethiopia, 2023

Variables		Minimum	Maximum	Mean	Std. Deviation
Compassion satisfaction		10.00	47.00	26.3366	9.37596
Compassion Fatigue		10.00	46.50	27.6290	9.40030
	BURNOUT	10.00	43.00	27.7936	9.63990
	Secondary traumatic stress	10.00	50.00	27.4644	9.85876

The findings of our study indicate that 258 (63.4%%) of the participants were found to have low levels of compassion satisfaction. Our study finding indicates the level of burnout and secondary traumatic stress is mainly average with 271(66.6%) and 266(65.4) respectively as shown in Table 3.

**Table 3:** Professional quality of life levels of nurses working at tertiary level hospitals in central Ethiopia, 2023

Variables		Low	Average	High
Compassion satisfaction		146(35.9%)	258(63.4%)	3(0.7%)
Compassion fatigue		124(30.5%)	275(67.6%)	5(0.5%)
	BURNOUT	131 (32.2)	271(66.6%)	5 (1.2%)
	Secondary traumatic stress	134 (32.9%)	266 (65.4%)	7 (1.7%)

### Compassion satisfaction

The statement “I am pleased with how I can keep up with helping techniques and protocols” had the highest agreement level in the study, with 153 responses (37.6%) indicating often and 127 responses (31.2%) indicating sometimes. The statement “I get satisfaction from being able to help people” has the lowest agreement level in the study, with 116 responses (28.5%) indicating Sometimes and 98 responses (24.1%) indicating Often. The statement “I feel invigorated after working with those I help” has the highest disagreement level in the study, with 153 responses (37.6%) indicating often and 71 responses (17.4%) indicating “never.” The statement “I believe I can make a difference through my work” had the lowest disagreement level in the study, with 85 responses (20.9%) indicating often and 2 responses (0.5%) indicating always as shown in (Table 4).

**Table 4:** compassion satisfaction of nurses working at tertiary level hospitals in central Ethiopia, 2023

Variables	Never		Rarely			Sometimes		Often		Always	
My work makes me feel satisfied.	152	37.3%		46	11.3%	147	36.1%	56	13.8%	6	1.5%
I like my work as a [helper]	123	30.2%		79	19.4%	142	34.9%	59	14.5%	4	1.0%
I am proud of what I can do to [help]	152	37.3%		46	11.3%	147	36.1%	56	13.8%	6	1.5%
I get satisfaction from being able to [help] people.	116	28.5%		61	15.0%	132	32.4%	98	24.1%	0	0.0%
I have happy thoughts and feelings about those I [help] and how I	144	35.4%		62	15.2%	133	32.7%	64	15.7%	4	1.0%
I feel invigorated after working with those I [help].	71	17.4%		36	8.8%	127	31.2%	153	37.6%	20	4.9%
I believe I can make a difference through my work.	82	20.1%		84	20.6%	154	37.8%	85	20.9%	2	0.5%
I am happy that I chose to do this work.	61	15.0%		37	9.1%	124	30.5%	162	39.8%	23	5.7%
I am pleased with how I can keep up with [helping] techniques and protocols	71	17.4%		36	8.8%	127	31.2%	153	37.6%	20	4.9%
I have thoughts that I am a “success” as a [helper].	93	22.9%		55	13.5%	143	35.1%	107	26.3%	9	2.2%

### Compassion fatigue

#### Secondary traumatic stress

The statement “I avoid certain activities or situations because they remind me of frightening experiences of the people I help” had the highest agreement level in the study, with 114 responses (28.0%) indicating often and 122 responses (30.0%) indicating sometimes. The statement “I feel as though I am experiencing the trauma of someone I have helped” has the lowest agreement level in the study, with 136 responses (33.4%) indicating often and 70 responses (17.2%) indicating never. The highest level of disagreement in the study was The statement “I feel depressed because of the traumatic experiences of the people” has the highest disagreement level, with 152 responses (37.3%) indicating Sometimes and 56 responses (13.8%) indicating Often. “The statement with the lowest agreement levels in the study was, “I think that I might have been affected by the traumatic stress of those I help.” has the lowest disagreement level, with 53 responses (13.0%) indicating often and 2 responses (0.5%) indicating always as shown in (Table 5).

**Table 5:** secondary traumatic stress of nurses working at tertiary level hospitals in central Ethiopia, 2023

Variables	Never		Rarely			Sometimes		Often		Always	
I feel depressed because of the traumatic experiences of the people	152	37.3%		46	11.3%	147	36.1%	56	13.8%	6	1.5%
I think that I might have been affected by the traumatic stress of those I [help]	119	29.2%		74	18.2%	159	39.1%	53	13.0%	2	0.5%
Because of my [helping], I have felt “on edge” about various things.	141	34.6%		62	15.2%	131	32.2%	70	17.2%	3	0.7%
As a result of my [helping], I have intrusive, frightening thoughts.	63	15.5%		42	10.3%	111	27.3%	160	39.3%	31	7.6%
I avoid certain activities or situations because they remind me of the frightening experiences of the people I	103	25.3%		62	15.2%	122	30.0%	114	28.0%	6	1.5%
I find it difficult to separate my personal life from my life as a [helper]	103	25.3%		62	15.2%	122	30.0%	114	28.0%	6	1.5%
I jump or am startled by unexpected sounds.	63	15.5%		48	11.8%	107	26.3%	167	41.0%	22	5.4%
I am preoccupied with more than one person I [help]	63	15.5%		42	10.3%	111	27.3%	160	39.3%	31	7.6%
I can’t recall important parts of my work with trauma victims.	103	25.3%		62	15.2%	122	30.0%	114	28.0%	6	1.5%
I feel as though I am experiencing the trauma of someone I have [helped].	70	17.2%		46	11.3%	103	25.3%	136	33.4%	52	12.8%

#### Burnout

The statement “I feel trapped by my job as a helper” has the highest agreement level in the study, with 132 responses (32.4%) indicating “Sometimes” and 98 responses (24.1%) indicating Often. The statement “I am the person I always wanted to be” had the lowest agreement level, with 152 responses (37.3%) indicating often and 43 responses (10.6%) indicating always. The highest disagreement levels in the study were The statement “I feel overwhelmed because my case workload seems endless” has the highest disagreement level, with 127 responses (31.2%) indicating often and 1 response (0.2%) indicating always. lowest disagreement levels, the statement “I feel happy” has the lowest disagreement level, with 152 responses (37.3%) indicating Sometimes and 56 responses (13.8%) indicating Often as shown in (Table 6).

**Table 6:** Burnout of nurses working at tertiary level hospitals in central Ethiopia, 2023

Variables	Never		Rarely			Sometimes		Often		Always	
I feel trapped by my job as a [helper].	116	28.5%		61	15.0%	132	32.4%	98	24.1%	0	0.0%
I feel “bogged down” by the system.	78	19.2%		48	11.8%	122	30.0%	148	36.4%	11	2.7%
I feel overwhelmed because my case [work] load seems endless	107	26.3%		53	13.0%	119	29.2%	127	31.2%	1	0.2%
I feel worn out because of my work as a [helper].	71	17.4%		36	8.8%	127	31.2%	153	37.6%	20	4.9%
I am not as productive at work because I am losing sleep over traumatic experiences of a person I [help]	109	26.8%		47	11.5%	90	22.1%	132	32.4%	29	7.1%
I am the person I always wanted to be.	82	20.1%		37	9.1%	93	22.9%	152	37.3%	43	10.6%
I am happy.	152	37.3%		46	11.3%	147	36.1%	56	13.8%	6	1.5%
I am a very caring person.	103	25.3%		62	15.2%	122	30.0%	114	28.0%	6	1.5%
I feel connected to others.	107	26.3%		53	13.0%	119	29.2%	127	31.2%	1	0.2%
I have beliefs that sustain me.	63	15.5%		42	10.3%	111	27.3%	160	39.3%	31	7.6%

### Job satisfaction

The study found that the overall mean score for job satisfaction among healthcare providers was 27.46 ± 9.86. This score falls within the moderate range of job satisfaction, indicating that healthcare providers in the study reported a moderate level of satisfaction with their jobs.

**Fig. 2:**
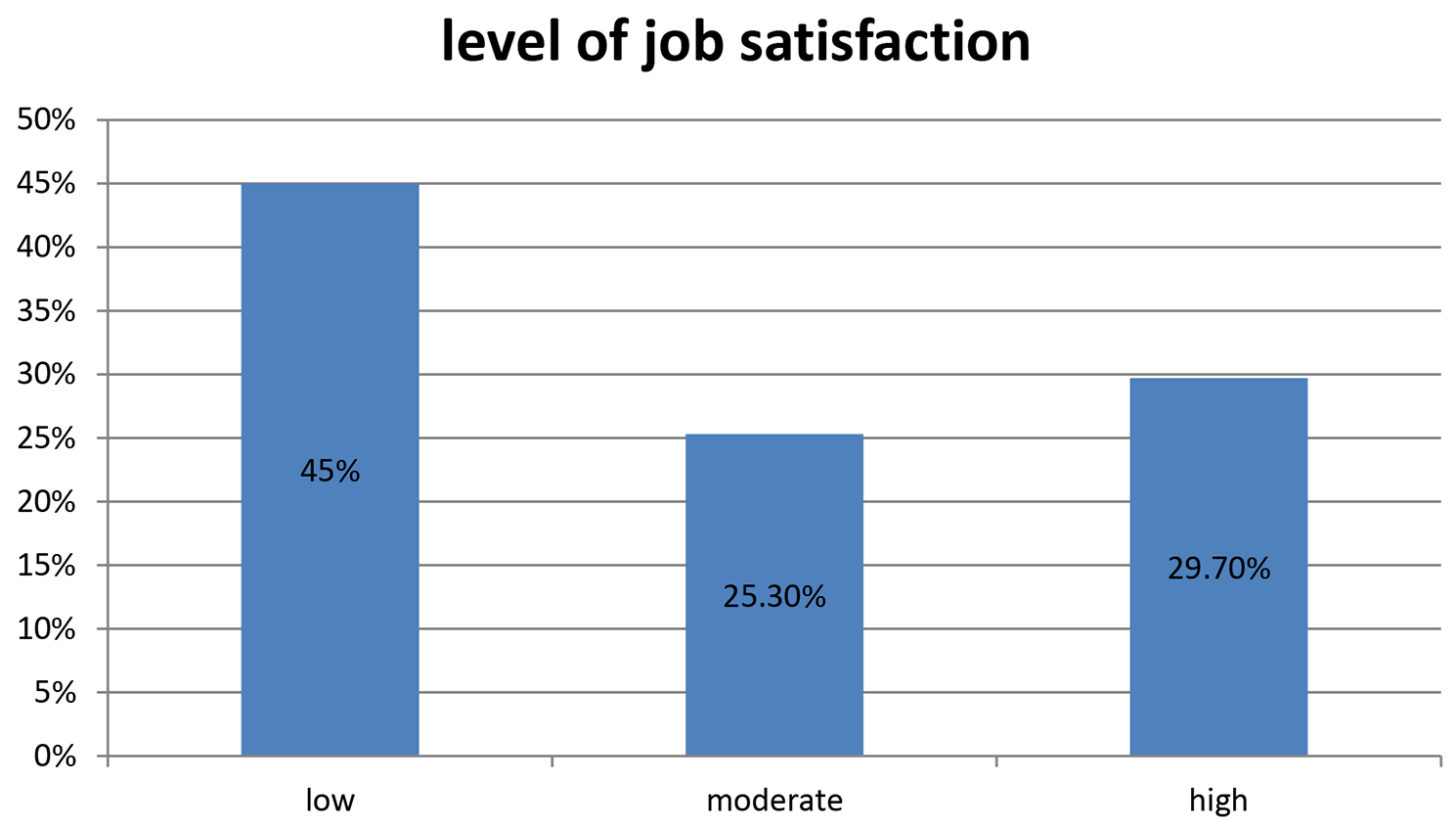
level of job satisfaction of nurses working at tertiary level hospitals in central Ethiopia, 2023

Furthermore, the study found that 55% of healthcare providers reported a moderate to high level of job satisfaction. There is still room for improvement, as the overall mean score for job satisfaction falls within the moderate range.

as shown in (Fig. 2).

Job satisfaction various conditions revealed that the nurses working in academic tertiary hospitals were satisfied with five factors: satisfaction in achievement (0.73449), advancement (1.4263 (0.69797), autonomy (1.6454 (0.69723), the recognition they get for good work (2.7232 (1.06691), leadership and organizational policy (2.2973 (0.96494), salary and working conditions (2.0511 (0.75966) and relationship (3.2973 (0.98667)) (Table 2). According to the mean score of each, the nurses working in academic tertiary hospitals were dissatisfied with the remaining fifteen factors. The highest level of dissatisfaction was reported for the opportunity for training or education at 1.36 (0.503). This was followed by a house allowance of 1.53 (0.749), hazard allowance for nurses of 1.93 (0.259), availability of resources and supplies of 2.17 (0.800), feeling about the job itself of 2.85 (1.319), and Salary (3.20 ± 1.535), respectively as shown in the (Table 7).

**Table 7:** job satisfaction of nurses working at tertiary level hospitals in central Ethiopia, 2023

	Strongly Disagree		Disagree			Neutral		agree		Strongly agree	
Achievement	91	22.4%	209	51.4%		88	21.6%	19	4.7%	0	0.0%
Advancement	270	66.3%	84	20.6%		46	11.3%	7	1.7%	0	0.0%
Autonomy	165	40.5%	162	39.8%		67	16.5%	13	3.2%	0	0.0%
Recognition	64	15.7%	66	16.2%		142	34.9%	119	29.2%	16	3.9%
Leadership	99	24.3%	108	26.5%		150	36.9%	44	10.8%	6	1.5%
Salary and working conditions	71	17.4%	147	36.1%		149	36.6%	40	9.8%	0	0.0%
Relationship	28	6.9%	22	5.4%	118		29.0%	169	41.5%	70	17.2%

### Correlation between compassion fatigue and job satisfaction

The finding of our study indicates compassion satisfaction and job satisfaction are strongly correlated, with a Pearson’s correlation coefficient of 0.762 and a *p*-value of 0.001. This indicates that there is a significant relationship between compassion satisfaction and job satisfaction among the nurses in the study. This strong correlation signifies a meaningful and significant relationship between compassion satisfaction and job satisfaction within the study cohort. This finding underscores the interconnectedness of these two factors and highlights the importance of addressing both compassion satisfaction and job satisfaction to enhance the overall well-being and job performance of nurses. as shown in the (Table 8).

**Table 8:** Pearson’s correlations (*p*-values) between variables

	Compassion satisfaction	Job satisfaction
Compassion satisfaction	1	0.762 (0.0001)*
Job satisfaction	0.762 (0.0001)*	1

Note: *p* < 0.001*

### Multiple linear regression analysis for compassion satisfaction and compassion fatigue

For Compassion fatigue, the results showed that marital status had a negative relationship with the outcome variable, with a standardized beta coefficient of − 0.127, indicating that participants who were not married had higher levels of compassion fatigue. The educational level also had a negative relationship with the outcome variable, with a standardized beta coefficient of − 0.269, indicating that participants with lower levels of education had higher levels of compassion fatigue. For compassion satisfaction, the results showed that marital status had a negative relationship with the outcome variable, with a standardized beta coefficient of − 0.132, indicating that participants who were not married had lower levels of compassion satisfaction. educational level also had a negative relationship with the outcome variable, with a standardized beta coefficient of − 0.244 and a, indicating that participants with lower levels of education had lower levels of compassion satisfaction as shown in (Table 9).

**Table 9:** Multiple linear regression analysis for compassion satisfaction and compassion fatigue

Variables	Unstandardized b	SE	standardized b	t	VIF
**Compassion fatigue constant**	32.064	2.630		12.190	1.029
Marital status	-2.378	0.856	− 0.127	-2.779**	1.153
Educational level:	-5.033	0.903	− 0.269	-5.573**	1.169
Experience in a year	2.400	0.889	0.131	2.698**	1.429
Income	− 0.421	0.092	− 0.245	-4.571	1.590
**Compassion satisfaction constant**	66.216	5.284		12.531	
Marital status	-4.964	1.720	− 0.132	-2.887**	1.029
Educational level:	-9.150	1.814	− 0.244	-5.043*	1.153
Experience in a year	4.689	1.787	0.128	2.624**	1.169
Income	− 0.907	0.185	− 0.263	-4.897	1.429

Compassion satisfaction R2 = 0.192, adjusted R2 = 0.181, F = 19.0, *p* < 0.001 Compassion fatigue R2 = 0.188, adjusted R2 = 0.178, F = 18.623,

** *p* < 0.001. *p < 0.01

### Multiple linear regression analysis job satisfaction

The results showed that educational level had a positive relationship with the outcome variable, with a standardized beta coefficient of 0.088 and a t-value of 2.712, indicating that participants with higher levels of education had higher levels of job satisfaction. Experience in a year had a negative relationship with the outcome variable, with a standardized beta coefficient of − 0.094 and a t-value of -2.740, indicating that participants with more years of experience had lower levels of job satisfaction as shown in (Table 10).

**Table 10:** Multiple linear regression analysis of job satisfaction

Variables	Unstandardized b	SE	standardized b	t
**Job satisfaction constant**	37.546	2.439		15.392
Sex	-0.495	0.720	-0.022	− 0.687
Marital status	0.596	0.767	0.027	0.777
Educational level:	1.908	0.703	0.088	2.712*
Experience in a year	-0.190	0.070	-0.094	-2.740*

R2 = 0.597, adjusted R2 = 0.591, F = 98.92, *p* < 0.001.

. *p < 0.01

## Discussion

This study was conducted to assess levels of professional quality of life and job satisfaction among nurses working at tertiary hospitals in central Ethiopia.

The finding of our study indicates that 64.1%% of the participants were found to have moderate to high levels of compassion satisfaction. Only 0.7% of the study participants have a high level of compassion satisfaction. This is lower than the study from China oncology nurses [36], 668, Guangzhou, Guangdong, China, 78% [37], a study from Nepal [38] with, a moderate level of compassion satisfaction is 71.3%, and a high-level compassion satisfaction 28.3%, Saudi Arabia, in which high of compassion satisfaction is 17.7% [35], a study from Thailand 75.3% [39], Northwest Ethiopia especially those on the high level of compassion satisfaction aspect in this study is 32.7% [27].

The discrepancies in studies can be attributed to various factors, including differences in sample size, for example, sample size from China oncology nurses [36], sample size 668, Guangzhou, Guangdong, China, its sample size 337 [37], and the specific population of nurses being studied.

This study indicated level of compassion fatigue is 124(30.5%), 275(67.6%), 5(0.5%) low, moderate and high level satisfaction respectively. Concerning components like burnout and secondary traumatic stress those who have moderate to high levels of burnout and secondary traumatic stress are 67%0.2,66.9% respectively.

The finding is consistent with a study from China on component burnout 63% and lower on secondary traumatic stress 76% component [37]. A similar study from a China frontline nurse in Wuhan [40] was higher than a study from Saud Arabia [35] in which the compassion fatigue level was 18% and burnout level 15%. Lower than the study from Nepal in which the level of compassion fatigue was moderate at 77% high level of 3.5% [38], a study from Uganda reported 49.11% high levels, 29.62% reported average levels, and 21.27% low levels of compassion fatigue [41]. Studies from oncology nurses also reported a high level of compassion fatigue and a low level of compassion satisfaction [34].

In our study, nurses working in central Ethiopia, have a moderate to high level of job satisfaction is 55%. This finding is consistent with other studies in Ethiopia like; a study from Jimma Ethiopia, with overall job satisfaction of [28], Bahir Dar Ethiopia [24], Ethiopia [42], and Ethiopian national pooled prevalence of job satisfaction [43], and lower than study from USA 2018 national sample survey of registered nurses, which 88.7% either extremely or moderately satisfied. Reasons for low level could be poor job conditions and limited resources [44], limited opportunities for career development, a combination of financial and non-financial incentives, and lack of motivation through incentives such as bonuses, house allowances, and salary increments [45].

The finding of our study indicates compassion satisfaction and job satisfaction are strongly correlated, with a Pearson’s correlation coefficient of 0.762 and a *p*-value of 0.001. This indicates that there is a significant relationship between compassion satisfaction and job satisfaction among nurses. This finding is in line with a study from the USA [8]. High levels of compassion satisfaction and job satisfaction can contribute to the overall well-being of nurses. Nurses’ satisfaction directly impacts the quality of care they provide to patients.

In this study, marital status had a negative relationship with the outcome variable, with a standardized beta coefficient of − 0.127 and a t-value of -2.779, indicating that participants who were not married had higher levels of compassion fatigue. The educational level also had a negative relationship with the outcome variable, with a standardized beta coefficient of − 0.269 and a t-value of -5.573, indicating that participants with lower levels of education had higher levels of compassion fatigue. This finding is consistent with a study from Turkey that found that being single or divorced and not having children were related to the highest levels of burnout in nurses [46].

The finding of our study indicates; that marital status and educational level were predictor variables for both compassion satisfaction and compassion fatigue.

This finding suggests that being married may serve as a protective factor against the development of compassion fatigue. This could be due to various reasons. For example, married individuals may have access to emotional support from their partners, which can help them cope with the emotional demands of their work. They may also have a stronger support system in general, including family and friends, which can contribute to their overall well-being and resilience. On the other hand, the negative relationship between educational level and compassion fatigue suggests that individuals with lower levels of education may be more susceptible to experiencing compassion fatigue. This could be attributed to several factors. For instance, individuals with lower levels of education may have limited access to resources and support systems that can help them cope with the emotional demands of their work. They may also have fewer opportunities for professional development and self-care, which can contribute to higher levels of emotional exhaustion.

For Compassion satisfaction, the results showed that marital status had a negative relationship with the outcome variable, with a standardized beta coefficient of − 0.132 and a t-value of -2.887, indicating that participants who were not married had lower levels of compassion satisfaction. educational level also had a negative relationship with the outcome variable, with a standardized beta coefficient of − 0.244 and a t-value of -5.043, indicating that participants with lower levels of education had lower levels of compassion satisfaction. This finding is consistent with systematic review and meta-analysis of oncology nurses [34].

The finding from our study indicates; that educational level has a positive relationship with nurses’ job satisfaction, with a standardized beta coefficient of 0.88. which indicates that participants with a higher level of education status had increased satisfaction with their jobs. On the other hand; experience in a year has a negative relationship with job satisfaction, with a standardized coefficient of -0.094 and a a t-value of -2.74. the finding was in line with study from Ethiopia [47] and Slovenia [34].

A study on the impact of broadcasting mistake management culture found that organizations that prioritize the quality of work life (QWL) of their personnel, which can include factors like educational opportunities, tend to have higher job satisfaction levels [48]. Another study on nursing home residents found that having more choice and control over relocation was associated with higher psychological well-being, which can be related to job satisfaction [49].

## Conclusion and recommendations

The study aimed to assess the levels of professional quality of life and job satisfaction among nurses working at tertiary hospitals in central Ethiopia. The majority of participants reported a medium level of compassion satisfaction, with a significant proportion experiencing moderate to high levels of compassion fatigue. Although more than half of the participants had moderate to high job satisfaction, there were still low levels of satisfaction. The study also identified predictors for compassion satisfaction and compassion fatigue, such as marital status, education, and experience, providing valuable insights into factors influencing professionals’ well-being. The strong correlation between compassion satisfaction and job satisfaction underscores the interconnectedness of these factors in nursing. Addressing the factors that contribute to satisfaction is essential for promoting the well-being of nurses and ensuring high-quality patient care. The study recommends developing targeted training programs, implementing workplace policies, and designing initiatives to enhance education, experience, and compassion satisfaction.

### Strength and limitation

The results of this study provide fresh knowledge and reflect the state of nurse professional quality of life and job satisfaction in tertiary hospitals of central Ethiopia. The sample was taken randomly, which nearly represented the population under the study. However, this study employed a cross-sectional study design and it was not possible to ascertain the temporal relationships between the outcome and explanatory variables.

## Data Availability

The dataset used and analyzed during the current study will be available from the corresponding author upon reasonable request.

## Abbreviations and Acronyms

CS: Compassion Satisfaction
CF: Compassion Fatigue
PQoL: Professional Quality of Life
AOR: Adjusted OD Ratio
CL: Confidence Interval
